# A Large-Scale Allosteric Transition in Cytochrome P450 3A4 Revealed by Luminescence Resonance Energy Transfer (LRET)

**DOI:** 10.1371/journal.pone.0083898

**Published:** 2013-12-23

**Authors:** Elena V. Sineva, Jessica A. O. Rumfeldt, James R. Halpert, Dmitri R. Davydov

**Affiliations:** 1 Skaggs School of Pharmacy and Pharmaceutical Sciences, University of California San Diego, La Jolla, California, United States of America; 2 Department of Biochemistry and Molecular Biology, Pennsylvania State University, University Park, Pennsylvania, United States of America; 3 Department of Chemistry and Biology, University of Waterloo, Waterloo, Ontario, Canada; Instituto de Tecnologica Química e Biológica, UNL, Portugal

## Abstract

Effector-induced allosteric transitions in cytochrome P450 3A4 (CYP3A4) were investigated by luminescence resonance energy transfer (LRET) between two SH-reactive probes attached to various pairs of distantly located cysteine residues, namely the double-cysteine mutants CYP3A4(C64/C468), CYP3A4(C377/C468) and CYP3A4(C64/C121). Successive equimolar labeling of these proteins with the phosphorescent probe erythrosine iodoacetamide (donor) and the near-infrared fluorophore DY-731 maleimide (acceptor) allowed us to establish donor/acceptor pairs sensitive to conformational motions. The interactions of all three double-labeled mutants with the allosteric activators α-naphthoflavone and testosterone resulted in an increase in the distance between the probes. A similar effect was elicited by cholesterol. These changes in distance vary from 1.3 to 8.5 Å, depending on the position of the donor/acceptor pair and the nature of the effector. In contrast, the changes in the interprobe distance caused by such substrates as bromocriptine or 1-pyrenebutanol were only marginal. Our results provide a decisive support to the paradigm of allosteric modulation of CYP3A4 and indicate that the conformational transition caused by allosteric effectors increases the spatial separation between the beta-domain of the enzyme (bearing residues Cys_64_ and Cys_377_) and the alpha-domain, where Cys_121_ and Cys_468_ are located.

## Introduction

With the increasing number of P450 structures that demonstrate conformational flexibility [1], [2], [3], [4], conformational changes accompanying enzyme-substrate interactions are recognized as a common feature that contributes to the ability of drug-metabolizing cytochromes P450 to metabolize a vast range of substrates [5], [6]. In addition to the role in adaptation of the P450 active site to substrates of various shapes and sizes, ligand-induced conformational rearrangements in some cytochromes P450 are hypothesized to be a core component of an allosteric mechanism that regulates the function of the microsomal drug-metabolizing ensemble [7], [8], [9]. This mechanism is thought to reveal itself in multiple instances of heterotropic cooperativity (activation of metabolism of one substrate by a second) observed with some drug-metabolizing P450s, of which human P450 3A4 (CYP3A4) is the most prominent.

Important support of allosteric modulation of CYP3A4 is provided by recent observations of a peripheral ligand binding site in the enzyme [10], [11], [12]. Interactions of this site located at the distal surface of the enzyme and surrounded by the F/F′ and G/G′ loops [10], [12] were hypothesized to be involved in CYP3A4 activation by steroids, α-naphthoflavone (ANF), and other heterotropic activators [11], [12], [13].

This mechanism suggests that allosteric effectors induce a conformational rearrangement that facilitates the formation of a catalytically competent enzyme-substrate complex [14], [15], [16], [17], [18]. Our recent design of a P450-compatible luminescence resonance energy transfer (LRET) donor-acceptor pair consisting of the phosphorescent dye erythrosine iodoacetamide (ERIA) and near-infrared fluorophore DY-731 maleimide (DYM) [13] provided a powerful tool for detecting these effector-induced conformational changes and probing their nature. The approach followed in this study is based on site-directed incorporation of the above pair of fluorophores which was enabled through design of three cysteine-depleted mutants of CYP3A4 possessing only two SH groups accessible to modification. The positions of the probes were selected at distant loci (distance between sulfur atoms ≥30 Å) in relatively rigid regions of the protein to ensure optimal sensitivity of LRET to large scale conformational rearrangements. The above design allowed us to probe the conformational response of the double-labeled protein variants to ligands exemplifying non-cooperative interactions, homotropic cooperativity, or heterotropic modulation of the enzyme.

The set of ligands used in this study included a high-affinity substrate bromocriptine, which does not reveal any cooperativity with 3A4 [15], [19], and 1-pyrenebutanol (1-PB), the interactions of which with the enzyme are characteristic of homotropic cooperativity [19], [20]. We also studied the effect of ANF and testosterone, which are known to cause a multifold increase in the enzyme turnover (heterotropic activation) [21], [22], [23], [24]. We also included in our set cholesterol, which is known to be both a substrate and a non-competitive inhibitor of CYP3A4 [25]. The basic premise was that this comparison will allow us to substantiate the allosteric mechanism of CYP3A4 modulation by ANF and testosterone through detecting a conformational rearrangement specific to the interactions of CYP3A4 with these heterotropic activators.

Our results demonstrate that the interactions of CYP3A4 with its modulators increase the spatial separation between the region of α-helix A and beta bundle (“beta-domain”) from the heme-containing core (“alpha-domain”) of the enzyme. In contrast, no considerable changes in the interprobe distance were elicited by bromocriptine or 1-pyrenebutanol. Besides providing strong support for the involvement of large scale conformational mobility in the mechanisms of heterotropic cooperativity in CYP3A4, our results provide the first information as to the structural nature of the effector-induced conformational changes.

## Materials and Methods

### Materials

ERIA was from AnaSpec. DYM was made by Dyomics and obtained from MoBiTec. 1-PB and tris(2-carboxyethyl)phosphine (TCEP) were from Invitrogen/Molecular Probes. ANF was the product of Indofine Chemical Company. Bromocriptine mesylate, Igepal CO-630 and cholesterol were obtained from Sigma-Aldrich. 3-Bromo-3-methyl-2-[(2- nitrophenyl)thio]-3H-indole (BNPS Skatole) was the product of Soltec Ventures. All other chemicals were of the highest grade available from commercial sources and were used without further purification.

### Site-directed mutagenesis and protein purification

The cysteine-depleted mutants of CYP3A4 were generated using the QuikChange site-directed mutagenesis kit and a template consisting of the cDNA of the N-terminally truncated (Δ3-12) CYP3A4 S18F variant with a tetrahistidine tag attached at the C-terminus (this variant of the wild-type enzyme that has a shortened N-terminal transmembrane helix is referenced in this paper as the “wild type CYP3A4” to distinguish that from the cysteine-depleted mutants), as described earlier [18]. Proteins were expressed in *E. coli* TOPP3 cells [26] and purified as described earlier [27].

### Modification with thiol-reactive probes

Prior to modification with thiol-reactive probes we eliminated TCEP contained in the storage buffer by two repetitive dilution/concentration cycles. Dilution (1∶10 v/v) with argon-saturated 100 mM Na-Hepes buffer, pH 7.4, containing 10% glycerol and 150 mM KCl (buffer A) was followed with concentration on a Centrisart I MWCO 100 kDA concentrator (Sartorius AG). Sequential labeling of double-cysteine mutants with two fluorescent probes was started with incubation of a 10 µM solution of protein with 10.5 µM ERIA or DYM (depending on desired order of labeling) in buffer A with constant stirring under an argon atmosphere at 25°C. The process of modification by ERIA was monitored by the decrease in the fluorescence of the label due to FRET to the heme. Modification by DYM was controlled by taking 50 µM aliquots of the reaction mixture and measuring their absorbance spectra after removal of unreacted label on BioSpin-6 spin columns (BioRad). After ∼95% completion of the reaction, the mixture was supplemented with 12 µM of the second label and the incubation continued for another 2 hours. The progress of the modification was controlled as described above. The reaction was terminated by addition of DTT to the final concentration of 3 mM. The DTT adducts of unreacted probes were removed from the concentrated samples by incubation with Bio-Beads SM-2 (Bio-Rad) followed by gel filtration on a Bio-Gel P6 (Bio-Rad) column equilibrated with buffer A.

The stoichiometry of labeling was determined based on UV-Vis absorbance spectra using the extinction coefficients of 0.088 µM^−1^cm^−1^ at 540 nm [28] and 0.24 µM^−1^cm^−1^ at 736 nm (according to MoBiTec documentation provided with the DY-731 dye) for ERIA and DYM, respectively.

### Fragmentation of the double-labeled C64/C468 mutant and analysis of the distribution of labels

Cleavage of the protein at tryptophan residues was performed with the use of BNPS-skatole ((3-bromo-3-methyl-2-[(2-nitrophenyl)thio]-3H-indole) [29], [30]. Specifically, 100 µL of 50–100 µM protein was mixed with 100 µL of acetic acid, and 600 µL of 1.3 mg/mL BNPS-skatole in acetic acid was added. The mixture was incubated at 45°C for 1 hour. To extract excess BNPS-skatole 800 µL of ethyl acetate and 1.6 ml of water were sequentially added to the reaction mixture and the sample was centrifuged at 2,000 rpm for separation of the phases. The water phase was collected and, after adjusting its pH to 7.5–8 with addition of NaOH, passed through a 20 kDa MWCO Centrisart-I concentrator (Sartorius AG) to remove undigested protein along with the largest (32 kDa) fragment and the products of partial fragmentation. The sample containing the N-terminal (7 kDa) and C-terminal (12 kDa) fragments was then concentrated to ∼300 µl on a VivaSpin-2 concentrator, MWCO 5 kDa (Sartorius AG). The distribution of the labels between the C-terminal peptide (that contains the tetra-histidine tag and the Cys_468_ residue) and the N-terminal fragment (that contains Cys_64_) was determined by comparing the spectra of absorbance of the sample before and after the passage through a 0.5 ml column of Ni-NTA Agarose.

### Spectroscopic measurements

All absorbance and fluorescence assays (including the time-resolved fluorescence measurements) were performed at a protein concentration ≤1 µM at 25°C with continuous stirring. Absorbance spectra were recorded with an S2000 rapid scanning CCD spectrometer (Ocean Optics). Ligand binding assays were done in 100 mM Na-Hepes buffer, pH 7.4, containing 0.5 mM EDTA. Titration experiments were performed with the use of 10–15 mM stock solutions of ligands in acetone, except for bromocriptine, which was used as a 300 µM solution in Na-acetate buffer, pH 4.0.

Fluorescence-based ligand binding assays were done under anaerobic conditions. Anaerobiosis was achieved using an oxygen-scavenging system consisting of protecatechuate-3,4-dioxygenase (0.5 units/ml) and 5 mM protocatechuic acid. The steady state and time-resolved phosphorescence measurements were performed with the use of a Cary Eclipse fluorescence spectrophotometer (Varian/Agilent Technologies). The excitation wavelength was set to 540 nm. Spectra were taken from 650 to 860 nm with 100 µs delay.

The steady-state luminescence emission anisotropy spectra were recorded with an Edinburgh Instruments FLS920 spectrofluorometer (Edinburgh Instruments Ltd.) equipped with a 6427 Xe flash lamp light source (Oriel Instruments/Newport Corporation), customary installed broadband polarizing beamsplitter cubes (CVI Melles Griot), and a thermostated cell holder. The correction factor (*I_HH_/I_HV_*), accounting for the difference in response of the detector to horizontally and vertically polarized light, was determined with the same samples.

### Data processing and analysis

Absorbance spectra obtained in titration experiments were analyzed with a principal component analysis (PCA) procedure complemented with least-square approximation of the principal vectors with a combination of the prototypical spectra of the CYP3A4 low-spin, high-spin and Type II-substrate-bound ferric CYP3A4 P450 states as described [19], [31].

Quantitative interpretation of LRET results was also based on the use of PCA applied to a series of emission spectra normalized on integral intensity. Approximation of the first principal vector (>98% of the observed changes) with a combination of the prototypical spectra of emission of ERIA and DYM allowed us to resolve the changes in the integral intensities of emission of each of the dyes. Intensity of the donor and acceptor fluorescence obtained in this way were used in determination of the efficiency of steady-state LRET using the equation 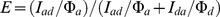
(Eq.1)where *I*_ad_ is emission intensity of the acceptor, *I*_da_ is the residual donor emission in the presence of acceptor (*I*_da_), *Φ*_a_ and *Φ*_d_ are the quantum yield of the fluorescence of acceptor and the quantum yield of the phosphorescence of donor, respectively [32].

The quantum yield of the DYM fluorescence was determined by the reference method, using a solution of Cy5 (*Φ* = 0.4) in water [32] as a fluorescent standard. The quantum yield of the ERIA phosphorescence was determined by comparison with the intensity of ERIA fluorescence and assuming the fluorescence quantum yield of ERIA to be equal to 0.2 [28]. The values of the phosphorescence quantum yield of ERIA and the fluorescence quantum yield of DYM for the protein-bound probes were estimated to be equal to 3.5·10^−3^ and 1.2·10^−2^, respectively.

The treatment of the time-resolved decay traces was performed using a bi-exponential approximation. The efficiency of time-resolved LRET was calculated using the equation
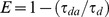
(Eq.2)


In this equation τ_da_ and τ_d_ designate the average lifetime of the excited state of the donor in the presence and absence of the acceptor, respectively. These average lifetimes are determined as τ = α_1_τ_1_+α_2_τ_2_, where τ_1,2_ and α*_1,2_* stay for the lifetime and the relative amplitude of each of the two exponential terms. In our calculations of the lifetime in double-labeled C64/C468, C377/C468 and C64/C121 proteins we used the lifetimes of phosphorescence measured with the respective proteins labeled with ERIA at a label:protein ratio 2∶1 as the estimates of τ_d_.

Distances were calculated using the equation:
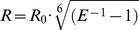
(Eq.3)where *R*_0_ is the Förster distance, and *R* is the distance between the donor and acceptor. *R*_0_ was calculated with the use of PhotoChemCad software [33]. In these calculations, we used the values of the orientation factor (*κ^2^*) and the refractive index (*n*) of 0.667 and 1.4, respectively.

To analyze the results of LRET titrations and the absorbance titration experiments with substrates revealing no homotropic cooperativity we used the equation for the equilibrium of bimolecular association ([34], p 73, eq. II-53):
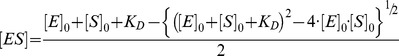
(Eq.4)


When fitting the results of titration experiments, where the changes in the monitored signal *A*_S_ (fraction of the enzyme in the high-spin state, intensity or the lifetime of the donor phosphorescence at a given substrate concentration) were assumed to be proportional to [*ES*], this equation was complemented with the offset *A*_0_ and the scaling factor *A*_max_ to yield the following relationship:
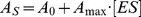
(Eq.5)


In those cases where the results of the absorbance titration experiments indicated homotropic cooperativity in the enzyme-substrate interaction (ANF, 1-PB and testosterone), we approximated the binding isotherms with the Hill equation.

All data treatment procedures and curve fitting were performed using a 32-bit version of our SPECTRALAB software [35] running under Windows XP™.

## Results

### Design of a LRET-based method for detecting ligand-induced conformational changes in CYP3A

An important aspect specific to the use of techniques based on RET (resonance energy transfer) to study conformational flexibility in cytochromes P450 is the absorbance of the heme chromophore. Selection of a FRET (fluorescence RET) donor/acceptor pair applicable in the studies of heme-containing proteins is challenging due to overlap of the heme absorbance with emission bands of most commonly used fluorescent probes. Furthermore, accurate measurement of distances with FRET requires thorough resolution of the excitation and emission bands of the donor and acceptor probes. An effective approach to deal with these challenges is provided by the use of LRET (luminescence RET) where the use of a phosphorescent donor with long-lived emission and registration of the delayed fluorescence ensures a thorough selectivity in monitoring of the emission that originates from energy transfer.

The majority of biophysical studies using LRET employ ligand-sensitized coordination complexes of lanthanide cations, usually europium or terbium, as phosphorescent donors [36]. These complexes exhibit lifetimes of phosphorescence in the range of 0.1–5 msec and are typically excited in the near-UV range [37]. However, the applicability of these probes in the studies of heme proteins is limited because of overlap of the excitation and emission bands of the probes with the absorbance spectra of the heme chromophore. Unambiguous data interpretation and accuracy of measurements in this case requires probes that emit in the far red and near infrared region, where the heme does not absorb. The use of the rare earth metals that emit in the near-infrared range (neodymium, ytterbium, or erbium) is, however, compromised by their much shorter lifetimes (<0.1 ms) and low quantum yield [37]. A viable alternative to the rare earth chelates is represented by a xanthene dye erythrosine, which possesses extensive phosphorescence at 695 nm. A very high quantum yield of the triplet state (*Φ*_T_ = 0.95 [38]) and its long lifetime (0.2–6 ms depending on environment [39]) make this dye potentially efficient as a LRET donor in biophysical applications.

These considerations prompted us to introduce a novel LRET donor/acceptor pair that consists of a phosphorescent probe erythrosine iodoacetamide (ERIA, donor) and a near-infrared fluorescent tandem dye DY-731 maleimide (DYM, acceptor) [13]. The structures of these thiol-reactive fluorophores are shown in Fig. S1. Considerable overlap of the spectrum of ERIA phosphorescence (λ_max_ = 695 nm) with the excitation band of DYM (λ_max_ = 736 nm) suggests a possibility of the Förster-type energy transfer between the probes. The *R*_0_ distance for this pair calculated using a value of the orientation factor (*κ* ^2^) equal to 2/3 (*i.e.*, assuming random rotation of at least one of the fluorophores) is equal to 34.6 Å. This new pair has been successfully used in our recent study of P450-P450 interactions in CYP3A4 [13].

### Selection of the points of attachment of the probes and construction of double-cysteine mutants

The large size of the fluorescent probes and relatively large Förster distance of the ERIA/DYM pair forced us to choose distant peripheral points of attachment for the probes. In order to minimize perturbations of the protein structure by mutagenesis we elected to use some of the native cysteine residues. CYP3A4 contains six potentially accessible cysteine residues - Cys_58_, Cys_64_, Cys_98_, Cys_239_, Cys_377_ and Cys_468_. However, Cys_58_ is known to have poor accessibility for modification [18] and cannot be used as a point of attachment. Cys_98_ and Cys_239_ are located in B/B′ loop and F′/G loops, respectively. These loops are known to belong to regions of high conformational flexibility [4], [40]. We therefore chose to refrain from placing the labels at these critical regions in order to minimize the effects of the probes on protein conformation.

**Figure 1:**
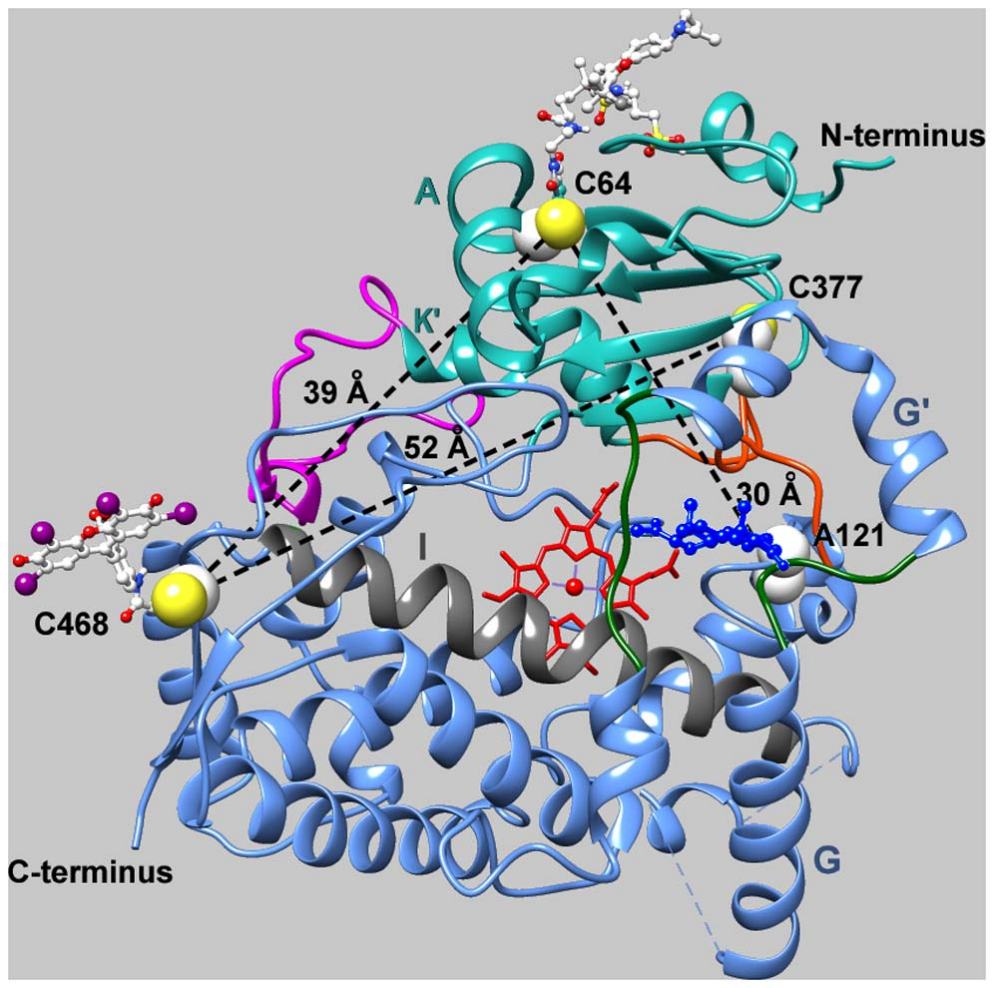
Position of the points of attachment of thiol-reactive probes used in this study. The cartoon representation of the structure of CYP3A4 is based on the coordinates of the enzyme complex with peripherally-bound progesterone (PDB 1W0F). Side chains of the cysteine residues C64, C377 and C468, as well as the alanine residue C121 (which we replaced with cysteine in our C64/C121 mutant) are shown in spheres. The structures of ERIA and DYM attached to residues Cys468 and Cys64 are shown in ball-and-stick representation. The part of the protein referred to here as the beta-domain is shown in sea green, while the alpha-domain is represented in blue (except for a-helix I, which is shown dark gray). The loops shown in magenta and orange are the meander loop and the B/B′ loop, respectively. The G/G′ and F/F′ loops, which surround the peripherally-bound molecule of progesterone (not shown), are shown in green. The molecule of progesterone bound in the proximity of the B/B′, F/F′ loops is shown as a dark-blue ball-and-stick model.

Two of the remaining three residues, namely Cys_64_ and Cys_377_, are located in the so-called beta-domain of the protein (the region of the beta-bundle, alpha-helices A and K′ and the N-terminal loop, Fig. 1). Due to the short distance between the sulfur atoms of these cysteines (19.8 Å), they cannot be used in a pair but may comprise the pairs with Cys_468_ instead. The latter residue is easily accessible to modification and is located in a loop between β_4_ strands in the C-terminal part of the protein. The high solvent exposure of this residue suggests a high degree of rotational mobility of the attached label, which simplifies the estimation of the interprobe distance in RET measurements. According to the X-ray structure of CYP3A4 (PDB 1W0F [10]) the distances from Cys_468_ to Cys_64_ and Cys_377_ determined from the X-ray structure are equal to 39 and 52 Å, respectively and lie in the range of reasonable sensitivity of the ERIA/DYM pair (*R*_0_ = 34.6 Å) to distance changes. Based on these considerations we selected Cys_64_/Cys_468_ and C_377_/C_468_ pairs for our studies. The respective mutants are designated in this article as C64/C468 and C377/C468 proteins.

In order to distinguish the concerted mobility of the beta-domain of the protein (where Cys_64_ and Cys_377_ are located) from local conformational changes in C-terminal loop (which bears Cys_468_) we decided to introduce another mutant where Cys_468_ is eliminated. In this case a non-native cysteine residue was introduced in a solvent-exposed position of the alpha-domain CYP3A4 through the A121C mutation. The distance from C64 or Cys377 to this newly-introduced cysteine is optimal for LRET measurements (∼30 Å). Due to the low yields of expression of CYP3A4(C64) and CYP3A4(C377) mutants, we decided to introduce A121C into a double-cysteine CYP3A4(C58/C64) mutant which yields much higher expression levels in bacteria. Due to poor accessibility of Cys_58_ [18], the modification of the resulting three-cysteine mutant is expected to take place at Cys_64_ and Cys_121_ positions only. This mutant we designate here as C64/C121 protein. The position of the pairs of cysteine residues designed in this way in the molecule of CYP3A4 is illustrated in Fig. 1.

### Properties of double- and triple-cysteine mutants of CYP3A4

Mutations introduced into CYP3A4 to eliminate its native cysteines were the same as described earlier [18]. As seen from Table 1, mutagenesis had a moderate impact on the parameters of interactions with the set of CYP3A4 ligands used in this study, namely 1-pyrenebutanol (1-PB), bromocriptine, α-naphthoflavone (ANF), testosterone, and cholesterol (the structures of these compounds may be found in Fig. S2. The minimal perturbation was observed with C64/C468, where the mutagenesis caused a small increase in the affinity and attenuated the cooperativity in the enzyme interactions with 1-PB, but had virtually no effect on the interactions with other substrates. In the case of C377/C468 and C64/C121 we observed some decrease in the affinity for 1-PB and bromocriptine and attenuation of homotropic cooperativity with 1-PB and ANF. In addition, these mutants exhibited decreased amplitude of the substrate-induced spin with all substrates. Aside from these variations, the basic characteristics of the wild type enzyme (the type of substrate-induced spectral changes, the pattern of homotropic cooperativity and relative affinity with different substrates, etc.) were not affected by mutagenesis (Table 1).

**Table 1:** Parameters of ligand-induced spin shift in CYP3A4 and its unlabeled and double-labeled mutants*.

Ligand	Protein	*K*_D_ or *S*_50_, µM	*ΔF*_HS_, %	*N_h_*
1-PB	W/T	9.5±0.9	50±1	1.6±0.4
	C64/C468	5.7±1.7	43±5	1.2±0.1
	C377/C468	14±2	29±9	1.2±0.2
	C64/C121	16±8	17±10	1.3±0.5
	C64/C468-ER/DY	5.4±2.1	18±2	1.3±0.1
Bromocriptine	WT	0.39±0.1	51±18	
	C64/C468	0.34±0.1	43±1	
	C377/C468	0.96±0.2	25±1	
	C64/C121	1.8±0.8	13±6	
	C64/C468-ER/DY	0.28±0.1	20±3	
	C64/C121-DY/ER	0.71±0.5	15±1	
	C377/C468-DY/ER	0.93±0.6	17±4	
ANF	WT	7.4±1.6	41±1	1.9±0.3
	C64/C468	5.1±1.5	39±8	1.8±0.2
	C377/C468	6.2±1.2	24±12	1.4±0.2
	C64/C121	8.6±1.6	19±4	1.3±0.4
	C64/C468-DY/ER	4.2±0.9	13±1	1.5±0.1
Testosterone	WT	43±6	47±1	1.1±0.1
	C64/C468	39±10	18±1	
	C377/C468	51±8	19±1	
	C64/C121	48±3	9±2	1.1±0.1
	C64/C468-ER/DY	38±6	27±5	
	C64/C121-DY/ER	60±2	19±3	
	C377/C468-DY/ER	33±10	22±4	

*The values given in the table represent the averages of 2–7 individual measurements, and the ± values show the confidence interval calculated for *p* = 0.05.

### Labeling of C64/C468, C377/C468 and C58/C64/C121 mutants with ERIA and DYM

**Figure 2:**
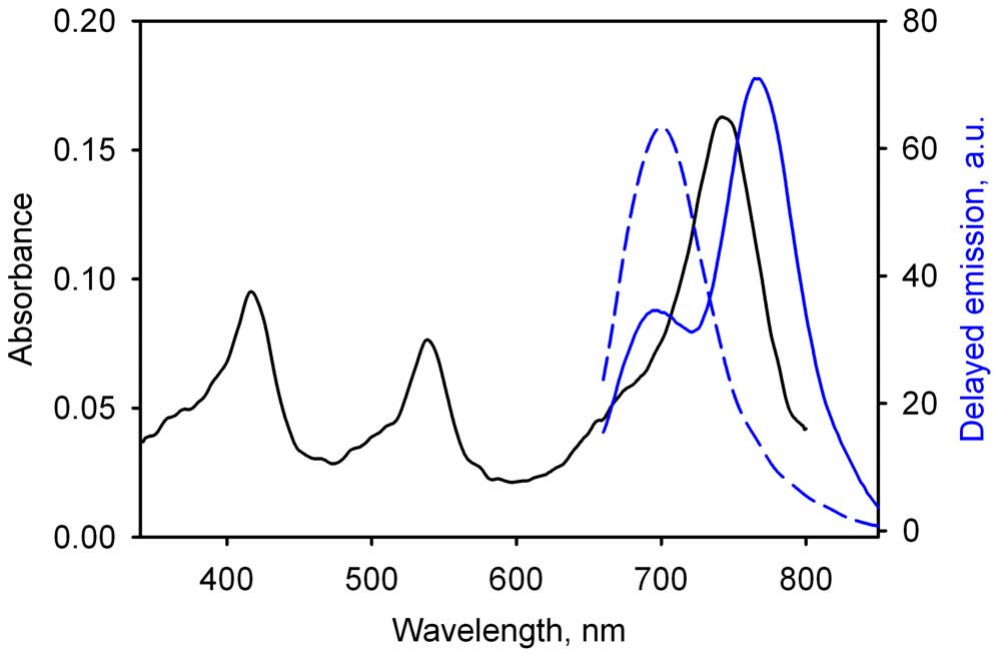
Spectra of absorbance and delayed fluorescence of the double-labeled C64/C468-ER/DY protein. Absorbance and fluorescence spectra of C64/C468-ER/DY are shown in solid black and solid blue lines respectively. Spectrum of delayed fluorescence of CYP3A4(C468)-ERIA is shown in blue dashed line.

In order to incorporate LRET donor/acceptor pair into our cysteine-depleted mutants we implemented a sequential labeling procedure (see *Experimental*), which resulted in preparations with near-equimolar stoichiometries of labeling by both labels. The spectrum of absorbance of the double-labeled C64/C468-ER/DY protein is exemplified in Fig 2.

Analysis of X-ray structures of CYP3A4 suggests that the Cys_468_ or Cys_121_ residues are the most exposed to solvent over the four points of label attachment used in our study. Therefore, we may expect that the label added first to C64/C468, C377/C468 and C64/C121 proteins will preferentially react with Cys_468_ or Cys_121_, while the second label will be preferentially located at Cys_64_ or Cys_377_. In the case of C64/C468 successively labeled with ERIA and DYM this inference was confirmed by fragmentation of the labeled protein with BNPS skatole, which cleaves at tryptophan residues [30]. Application of the fragmented protein to Ni-NTA agarose results in preferential retention of the ERIA-containing peptide, while most of the DY-731 label was found in the pass-through fraction. This result suggests that the predominant part of ERIA is attached to the C-terminal peptide containing both the Cys_468_ and the tetra-hystidine tag. According to our analysis the distribution of ERIA label between Cys_468_ and Cys_64_ residues is close to 3∶1 (see Fig. S3).

In order to minimize the steric constrains for incorporation of the labels we used the more bulky DYM label as the first modifying reagent in all cases except for C64/C468, where we compared two double-labeled derivatives with opposite orders of labeling. It should be noted also that our attempt to obtain C377/C468-ER/DY resulted in complete precipitation of the double-labeled protein. This observation confirms asymmetric distribution of the labels in double-labeled proteins and suggests that the modification of Cys_377_ by DYM is deleterious for the enzyme. The double labeled proteins obtained with DYM as the first modifying reagent are designated below as DY/ER derivatives, whereas the double-labeled C64/C468 obtained with the opposite order of labeling is designated as C64/C468-ER/DY.

The parameters of interactions of the double-labeled proteins with a set of CYP3A4 ligands are shown in Table 1. It can be seen that the effect of labeling on the parameters of interactions and the amplitude of substrate-induced spin shift were quite marginal. The only pronounced effect was a decrease in the amplitude of the ligand-induced spin shift observed in the interactions of labeled C64/C468 with all ligands (Table 1). However, this decrease had virtually no effect on the spin state of the final enzyme-substrate complexes due to the higher content of the high-spin state in the ligand-free C64/C468-ER/DY.

### LRET in double-labeled CYP3A4 mutants

As exemplified in Fig 2, labeling of C64/C468, C64/C468, and C377/C468 mutants with ERIA results in appearance of a band of erythrosine phosphorescence centered at 695 nm. As summarized in Table S1, the phosphorescence life time of the protein-bound ERIA in the ERIA-labeled C64/C468, C377/C468, and C64/C121 proteins where both cysteine residues are modified with the phosphorescent probe was 0.23, 0.18, and 0.28 ms (Table S1). These estimates are in a good agreement with the values of 0.18 and 0.32 ms reported for the lifetime of erythrosine phosphorescence in aqueous and ethanol solutions, respectively [39].

**Figure 3:**
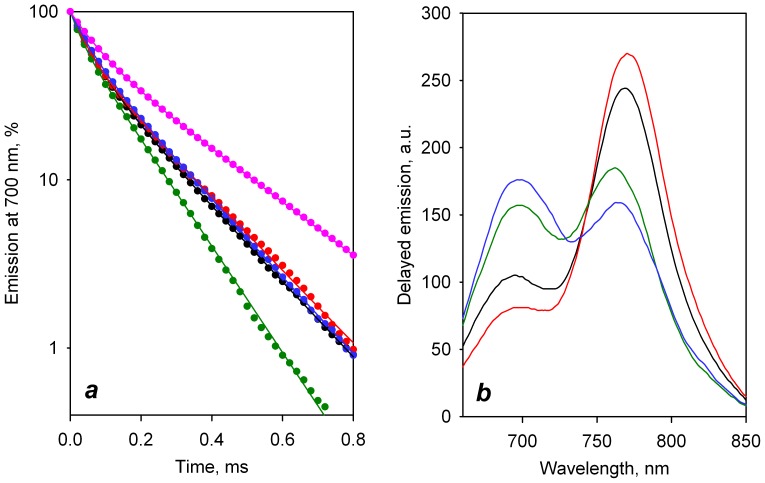
Spectra of delayed emission and the erythrosine phosphorescence decay traces of labeled derivatives of CYP3A4. Panel ***a*** shows the decay traces obtained with the double labeled C64/C468-ER/DY (black), C64/C468-DY/ER (red), C64/C121-DY/ER (green), C377/C468-DY/ER (blue),and the single-labeled CYP3A4(C468)-ERIA (magenta). Spectra of delayed emission shown in panel ***b*** represent C64/C468-ER/DY (black), C64/C468-DY/ER (red), C64/C121-DY/ER (green) and C377/C468-DY/ER (blue).

Consistent with the expectation of LRET in the ERIA/DY-731 pair, incorporation of these fluorophores with the production of the C64/C468-ER/DY, C64/C468-DY/ER, C377/C468-DY/ER and C64/C121-DY/ER derivatives results in a considerable decrease in the phosphorescence lifetime as compared to ERIA-labeled derivatives (Fig. 3a, Table S1). Along with the change in the lifetime, the spectra of delayed emission of the double-labeled derivatives exhibit an ample band of delayed emission of DYM, which is also indicative of the energy transfer between the probes (Fig. 3b). The fact that the appearance of the delayed fluorescence of the acceptor is accompanied by the commensurate changes in the donor lifetime suggests that the energy transfer between ERIA and DYM is nonradiative and therefore obeys either Förster (dipole-dipole coupling) or Dexter (direct electron transfer) mechanisms. However, the large distance between the probes in our constructs makes Dexter energy transfer very unlikely, as it requires an orbital overlap interaction between the donor and acceptor molecules and has an exponential dependence on the interprobe distance ([41], p. 411)

The estimates of the interprobe distance calculated from the results of our steady-state fluorescence and lifetime measurements assuming the Förster type of energy transfer are shown in Table 2. As seen from this table, given the large sizes of ERIA and DYM probes (10–12 and 15–20 Å respectively), these estimates are in good agreement with the distances between the sulfur atoms in the labeled proteins suggested by X-ray structure PDB 1W0F [10]. This fact, together with the reasonable agreement between the distance estimates found from the steady-state and lifetime measurements, justifies the above presumption that the energy transfer between ERIA and DYM obeys the Förster mechanism.

**Table 2:** Interprobe distances in the labeled CYP3A4 derivatives*.

		Steady-state fluorescence		Lifetime measurements	
Labeled protein	Distance in X-ray structure, Å^a^	*E*, %^b^	*R*, Å	*E*, %^b^	*R*, Å
(C64/C468)-ER/DY	39.0	29.3±4	39±2	42.8±3	36±1
(C64/C468)-DY/ER	39.0	44.7±2	35±2	41.1±5	37±1
(C377/C468)-DY/ER	51.8	15.3±1	45±1	40.7±2	37±1
(C64/C121)-DY/ER	30.0	19.4±1	43±1	48.0±3	35±1

*The values given in the table represent the averages of at least 10 individual measurements, and the ± values show the confidence interval calculated for *p* = 0.05.

a Distances observed in the PDB structure 1W0F. The distances were measured between the sulfur atoms of cysteines, except for C64/C121, where the value correspond to the distance between the sulfur atom of Cys_64_ and C-beta carbon of alanine (which is the native residue at this position).

b Efficiency of LRET. In the case of steady-state measurements these values were calculated from the spectra of delayed emission according to Eq. 1. For the lifetime measurements the calculations were done with Eq. 2 using the values of τ_d_ (lifetime in the absence of acceptor) determined with the respective mutants labeled with ERIA only (see Table S1.

To characterize the rotational mobility of the labels and estimate possible inaccuracy in the distance calculations caused by uncertainty in their orientation we studied the anisotropy of emission in single-labeled C468-ER and in double-labeled C64/C468-ER/DY. The anisotropy of phosphorescence of the single-labeled C468-ER at 700 nm was equal to 0.34±0.02. The depolarizing effect of LRET [42] observed in C64/C468-ER/DY causes a decrease in this parameter to 0.28±0.02 in the double-labeled protein. In contrast to the moderately polarized phosphorescence of ERIA, the fluorescence of the acceptor (DYM) was essentially depolarized. The low value of anisotropy of DYM fluorescence (0.07±0.02 at 780 nm) is indicative of high rotational mobility of this fluorophore, which justifies the use of the isotropic orientation factor (*κ* ^2^ = 2/3) in our calculations. The values of anisotropy derived from these measurements allowed us to assess uncertainty in our estimates of *R*_0_ caused by possible deviation of the orientation factor from the isotropic value. According to analysis with the method of Haas and co-authors [43], maximal possible deviation of the actual interprobe distance in C64/C468-ER/DY from the estimates obtained with LRET may range from −10 to +18%.

### LRET in mixed oligomers of single-labeled CYP3A4(C468)-ERIA and CYP3A4(C64)-DYM

Similar to other membranous P450 proteins, CYP3A4 in solution is known to form large oligomers ranging from 215 to 450 kDa [17]. Such oligomerization raised the possibility that the LRET observed in our studies might involve donor and acceptor molecules situated in different subunits of the oligomer (intermolecular LRET). In order to test this possibility we studied LRET in mixed oligomers formed of single-cysteine mutants CYP3A4(C468) and CYP3A4(C64) labeled with ERIA and DYM, respectively (C468-ER and C64-DY). To promote the formation of mixed oligomers we monomerized the equimolar mixture of these proteins by addition of 0.2% Igepal CO-630 [9], [17] and, after incubation for 30 min at 25°C, removed the detergent by incubation with Bio-Beads SM-2 (Bio-Rad) followed by gel filtration on a BioSpin-6 (Bio-Rad) column.

The efficiency of LRET observed in mixed oligomers did not exceed 5%, which corresponds to a donor-acceptor distance of >60 Å. This is in stark contrast to the values of 29% and 39 Å found with the double-labeled C468/C64-ER/DY (Table 2). The results indicate that in the latter case the predominant part of the observed signal is attributable to LRET between the fluorophores situated in the same protein molecule.

### Effect of substrates on LRET in double-labeled derivatives of C64/C468

**Figure 4:**
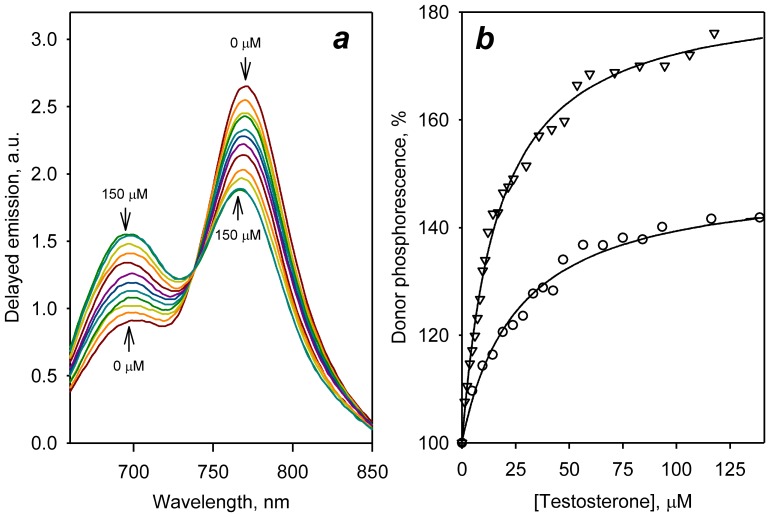
Titration of double-labeled derivatives of C64/C468 with testosterone monitored by steady-state fluorescence spectroscopy. (*a*) A series of the spectra of delayed emission of C64/C468-ER/DY recorded at increasing concentrations of testosterone; (*b*) Changes in the intensity of erythrosine phosphorescence in the titrations of C64/C468-ER/DY (circles) and C64/C468-DY/ER (triangles) with testosterone. The solid line represents the results of fitting of the data set with Eq. 4.

A representative series of spectra obtained in titration of C64/C468-ER/DY with testosterone is exemplified in Fig. 4A. Addition of testosterone results in an increase in the intensity of donor (ERIA) phosphorescence concomitant with a decrease in the emission of the acceptor (DYM). These changes demonstrate a pronounced decrease in LRET efficiency, which is indicative of a ligand-induced increase of the interprobe distance. Fig. 4B shows the titration curve obtained from the analysis of the dataset shown in Fig. 4A along with the curve obtained in the testosterone titration experiment with C64/C468-DY/ER. It can be seen that the change in the order of labeling of C64/C468 affected the amplitude of the observed changes, but did not change the shape of the titration curve.

Changes in spectra of delayed emission similar to those shown in Fig. 4 were also observed in the titrations of both labeled C64/C468 derivatives with ANF, a prototypical heterotropic activator. A pronounced effect was also observed with cholesterol, which has been shown to inhibit the activity of CYP3A4 in a non-competitive manner [25]. Importantly, the *K*_d_ values obtained in LRET titrations with these ligands (Table 3) are in good agreement with the studies of their binding to CYP3A4 monitored by UV-Vis absorbance spectroscopy using the ligand-induced spin shift (Table 1). This observation indicates that the ligand-induced conformational changes detected by LRET represent a consequence of specific interactions of the enzyme with ligands rather than being a result of some unspecific interactions of the ligands with the fluorophores.

**Table 3:** Parameters of ligand interactions with CYP3A4 determined by steady-state and time-resolved LRET*.

		Steady-state fluorescence			Lifetime measurements	
Protein/	Ligand	*K*_d_ (µM)	Δ*E,* %	Δ*R,* Å	Δ*E_LT_*,%	Δ*R,* Å
(C64/C468)-ER/DY	1-PB	N/D^a^	1±1	−0.3±0.8	−1.5±1	0.4±0.1
	ANF	17.3±7.2	−8±4	2.1±0.9	−14.5±1	4.1±0.2
	Bromocriptine	N/D	−1±1	0.5±1.6	+1.2±1	−0.3±0.6
	Cholesterol	7.7±0.9	−10±5	3.5±0.7	−16.6±1	4.8±0.2
	Testosterone	22.8±3.6	−9±4	3.1±1.0	−15.6±2	4.5±0.5
(C64/C468)-DY/ER	1-PB	22.2±4	−4±1	0.9±1.8	+3.8±1	−0.9±0.1
	ANF	9.1±2.9	−18±1	3.9±1.7	−6.8±3	1.8±0.7
	Bromocriptine	0.7±0.1	−2±1	0.2±0.2	+0.4±1	0.3±1.0
	Cholesterol	6.5±1.1	−11±1	5.9±2.0	−5.9±3	1.6±1.0
	Testosterone	16.4±3.5	−19±4	5.5±1.9	−16.1±3	4.9±0.5

*Life-time measurements were performed at nearly-saturating ligand concentrations, which were equal to 100 µM, 2.5 µM, 50 µM and 100 µM for ANF, bromocriptine, cholesterol, and testosterone, respectively. The respective values of LRET efficiency (Δ*E_LT_*) were calculated according to Eq. 2 using the value of τ_d_ (lifetime in the absence of acceptor) equal to 234 µs (see Table S1). In the case of steady state measurements, the change in LRET efficiency (Δ*E*) were calculated from the maximal relative change in the intensity of donor fluorescence found from the approximation of LRET titration curves with a combination of Eq. 4 and Eq. 5. The values given in the table represent the averages of 3–7 individual measurements, and the ± values show the confidence interval calculated for *p* = 0.05. The values of the distance changes were considered significant if their confidence interval falls outside the −0.5 - +0.5 Å window. These values are emphasized in bold.

a Not determined – in these cases the amplitude of the changes was too small to obtain a reliable estimate of the dissociation constant.

In contrast to the above apparent allosteric effectors, no significant changes in LRET efficiency in C64/C468-ER/DY were observed with the high-affinity substrate bromocriptine, which does not show any cooperativity with CYP3A4. Similarly, no ligand-induced changes were observed with 1-PB, a model CYP3A4 ligand and potential substrate that exhibits significant homotropic cooperativity [19], [20]. A similar picture was also observed with C64/C468-DY/ER. Here again, significant changes in the interprobe distance were observed with ANF, testosterone, and cholesterol only. Although the interactions of 1-PB and bromocriptine with C64/C468-DY/ER resulted in a marginal increase in LRET efficiency, the respective estimates of the changes in interprobe distances were statistically insignificant (Table 3).

**Figure 5:**
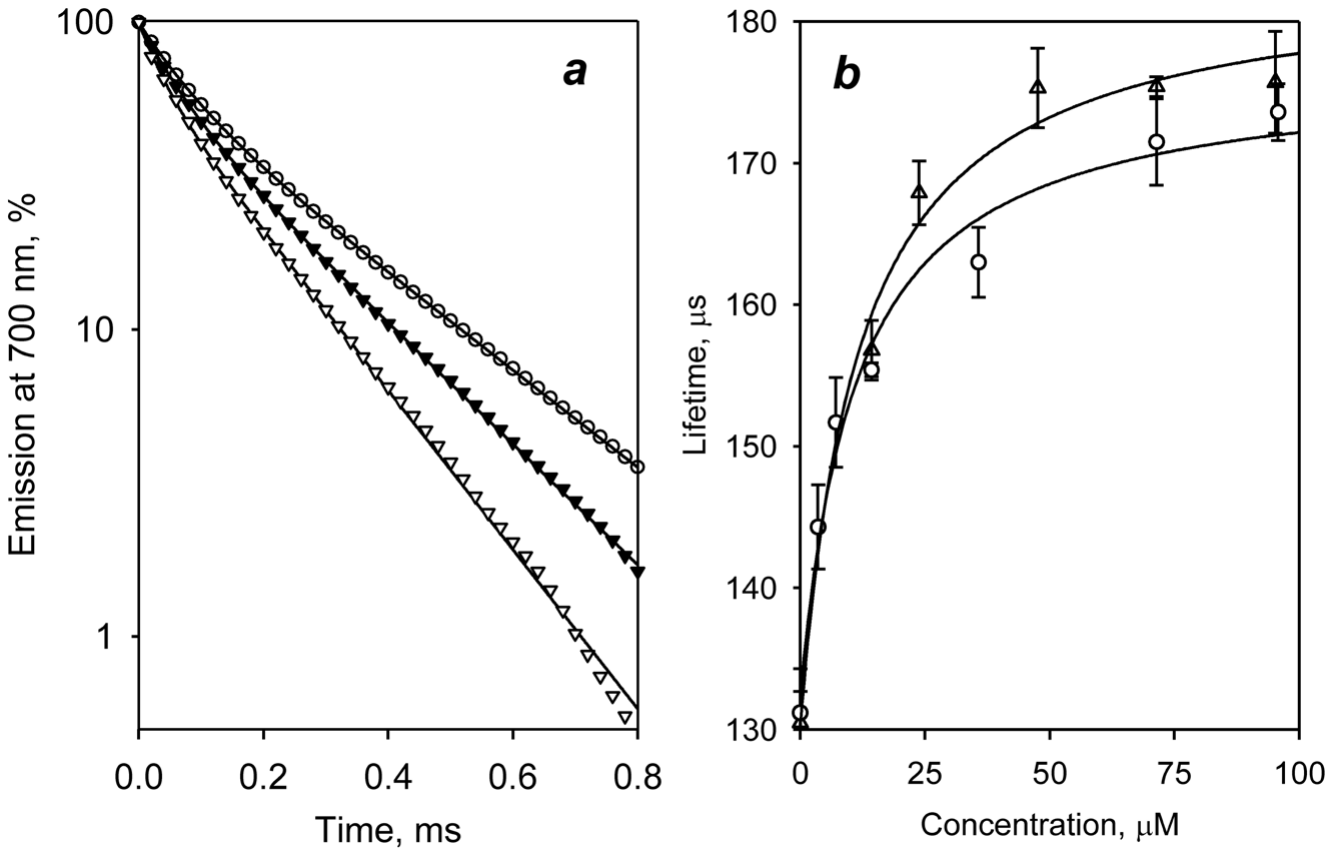
Titration of double-labeled derivatives of C64/C468-ER/DY with ligands monitored by lifetime measurements. (*a*) Phosphorescence decay traces obtained with CYP3A4(C468)-ERIA (circles) and C64/C468-ER/DY at no substrate added (open triangles) and in the presence of 100 µM testosterone (closed triangles). Solid lines represent the bi-exponential approximations (ρ^2^>0.999). (b) Dependence of the average lifetime of phosphorescence <τ_d_> in C64/C468-ER/DY on the concentration cholesterol (circles) and ANF (triangles). The lines show the results of fitting of the data sets to Eq. 4.

The effect of CYP3A4 ligands on interprobe distance in double-labeled C64/C468 derivatives was also confirmed in the time-resolved fluorescence measurements. Fig, 5A illustrates the effect of testosterone on the phosphorescence decay traces in these two labeled derivatives. The interactions of both labeled derivatives with testosterone result in a substantial increase in the lifetime of the donor fluorophore, which is consistent with the decrease in LRET efficiency observed in our steady-state titration experiments. Curves obtained from phosphorescence decay traces of C64/C468-ER/DY titrated with ANF and cholesterol are shown in Fig. 5B. The results of bi-exponential analysis of the decay traces are summarized Table S2.

As seen from data presented in Table 3, although there are some differences between the values of distance changes determined with the two methods, they are in a good qualitative agreement. Both methods reveal significant increases in the interprobe distance induced by ANF, testosterone, and cholesterol, while the changes upon the binding of 1-PB and bromocriptine were marginal. The good correlation between the results obtained with C64/C468-DY/ER and C64/C468-ER/DY derivatives suggest that the observed ligand-induced changes in interprobe distance are due to a large-scale conformational transition in the protein rather than being caused by some local rearrangements changing the orientation of the probes in an otherwise rigid protein molecule.

### Effect of ligands on LRET in double-labeled C377/C468 and C58/C64/C121 mutants

**Figure 6:**
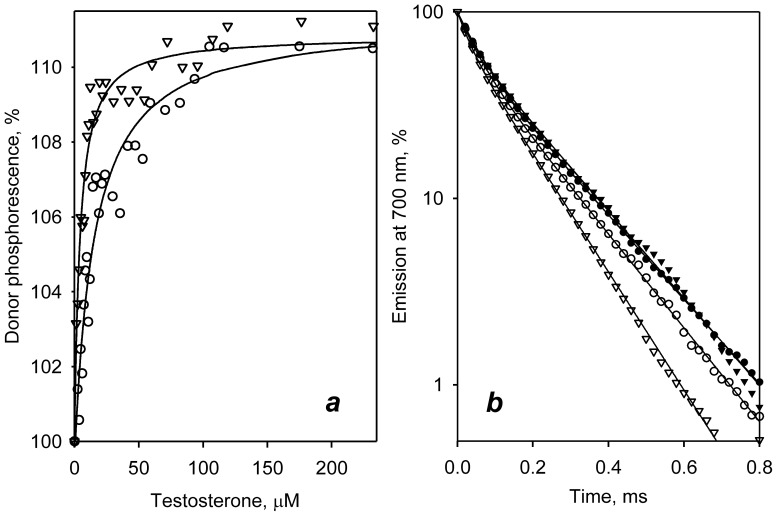
Interactions of double-labeled CYP3A4 proteins with testosterone monitored by LRET. Interactions of C377/C468-DY/ER (circles) and C58/C64/C121-ER/DY (triangles) with testosterone monitored by LRET in steady-state (*a*) and lifetime (*b*) setup. Panel *a* shows the changes in the intensity of donor fluorescence observed in titrations of the double-labeled proteins with testosterone. The lines show the results of fitting of the data sets to Eq. 4. Panel *b* shows the phosphorescence decay traces recorded in the absence of added ligand (open symbols) and in the presence of 100 µM testosterone (closed symbols)

The LRET experiments with C377/C468-DY/ER and C58/C64/C121-DY/ER revealed behavior similar to that depicted above for the double-labeled C64/C468 derivatives. Here again the enzyme interactions with testosterone, ANF, and cholesterol resulted in a decrease in LRET efficiency that suggests an effector-induced increase in spatial separation between the labeled residues. The effects of testosterone on the intensity and the lifetime of the donor phosphorescence are illustrated in Fig. 6. In contrast, the interactions of 1-PB with both C377/C468-DY/ER and C64/C121-DY/ER and binding of bromocriptine to C377/C468-DY/ER had virtually no effect on LRET monitored in either setup. However, both the steady-state and lifetime experiments revealed a statistically significant increase in the interprobe distance in C64/C121-DY/ER by 1.3 Å in response to the binding of bromocriptine. This effect of bromocriptine is characteristic for C64/C121-DY/ER only and was not observed with any other positions of the probes.

Ligand-induced changes in LRET efficiency with all four double-labeled derivatives of CYP3A4 in both steady-state and lifetime setup are summarized in Table S2. Interestingly, the correlation between the results obtained with the steady-state and lifetime setup with the C377/C468-DY/ER is much worse than that observed with three other mutants. While the changes detected in this protein with the lifetime approach were the highest among all four labeled derivatives, the steady-state setup revealed significant changes only in the presence of testosterone, whereas the effect of ANF and cholesterol was only marginal (see Table S2). This underestimation of ligand-induced changes by steady-state spectroscopy may be caused by modulation of the intensity of donor fluorescence by some mechanism other than LRET. Thus, an increase in donor phosphorescence caused by a ligand-induced decrease in LRET efficiency may be partially compensated in C377/C468-DY/ER due to attenuation of the quantum yield of the donor by some ligand-induced changes in its local environment. It should be noted in this context that the estimates of the interprobe distances deduced from the lifetime measurements are generally considered more robust and accurate than the values suggested by the steady-state spectroscopy [44].

**Figure 7:**
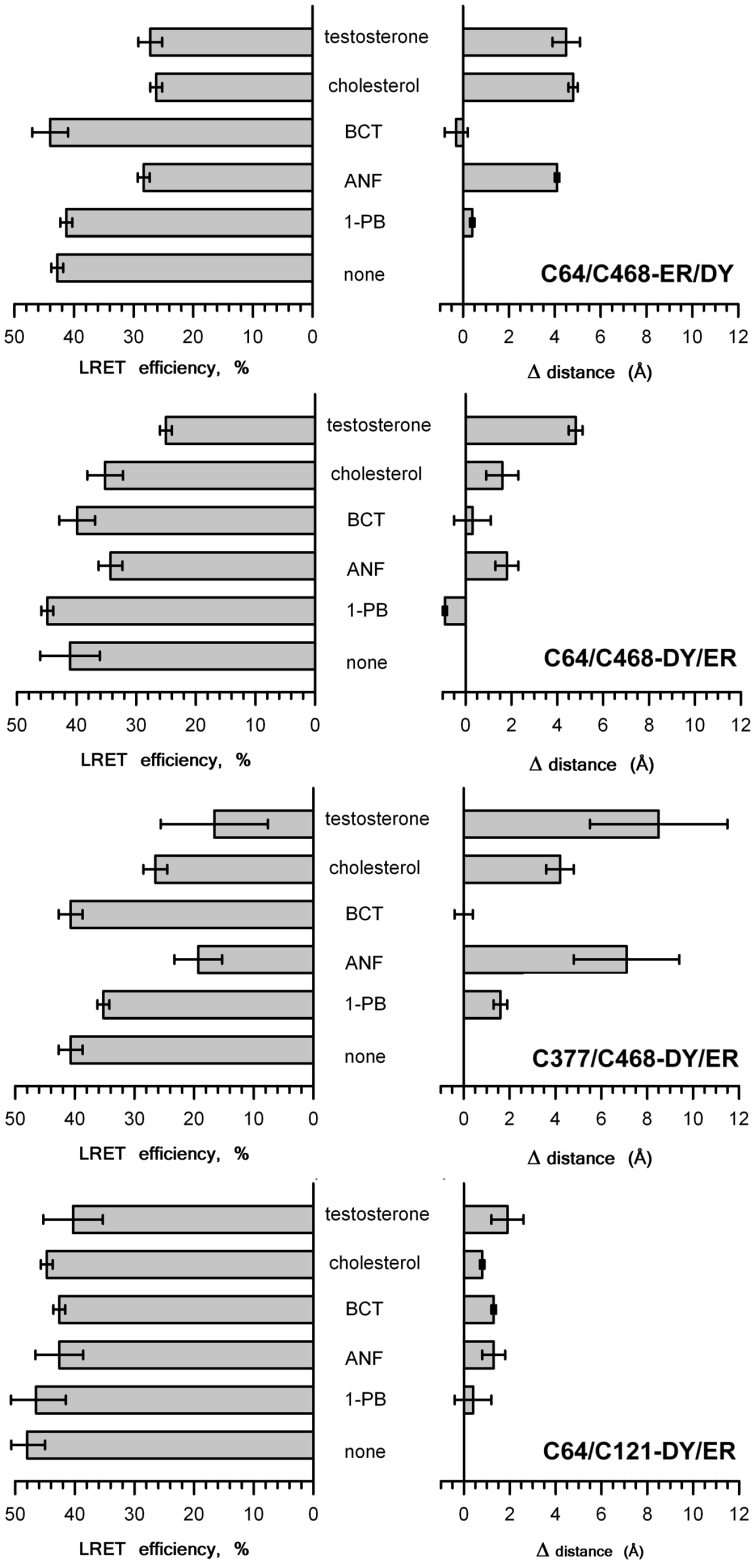
Effect of CYP3A4 substrates and allosteric effectors on efficiency of LRET (left) and interprobe distances (right) in double-labeled mutants.

The effect of ligands on LRET efficiency and the corresponding estimates of the changes in interprobe distance based on lifetime measurements with all four double-labeled derivatives are compared in Fig. 7. As seen from this figure, the general regularities in the effects of different ligands are quite similar for all three pairs of the points of label attachment.

## Discussion

The most important outcome of this study is the finding that the interactions of CYP3A4 with the activators of the enzyme ANF and testosterone result in a considerable increase in spatial separation between the labeled residues in all three probed pairs. Similar but less pronounced effect was elicited by cholesterol, which is known to be a non-competitive inhibitor of the enzyme. The largest increase (up to 8.4 Å) is elicited by testosterone, while the effect of cholesterol is the smallest in most cases.

It should be noted, however, that the ligand-induced distance changes determined in this study should not be thought of as direct characterization of distinct conformers specific to enzyme complexes with the respective compounds. According to the modern concepts of allostery, the native state of a protein is considered as a conformational ensemble, where allosteric perturbation involves a redistribution of pre-existing conformations [45], [46]. Thus, the estimates of the interprobe distance deduced from LRET measurements represent an average over the distances characteristic of multiple protein conformations weighted proportionally to their representation in the entire conformational ensemble. Therefore, our estimates of the ligand-induced distance changes should be rather considered as measures of a degree of displacement of conformational equilibrium by different ligands.

In contrast to ANF and testosterone and cholesterol, the substrates bromocriptine and 1-PB, which are not found to elicit any heterotropic activation in CYP3A4, do not induce any substantial changes in LRET efficiency in most cases. The only exception is C64/C121-DY/ER, where the addition of bromocriptine induces an increase in the interprobe distance by 1.3 Å, which is comparable with the changes caused by ANF, testosterone and cholesterol (Fig. 7). This peculiarity of C64/C121-DY/ER may reflect the location of Cys_121_ at the C-terminal end of the B′/C loop. Positioning of one of the fluorophores at the periphery of the flexible B/C block, which is known to undergo conformational rearrangements in enzyme-substrate interactions [40], may make the interprobe distance sensitive to local conformational changes in this region upon binding of a bulky bromocriptine molecule. It should be noted, however, that according to the X-ray structure of the enzyme complex with bromocriptine (PDB 3UA1), the overall conformational changes induced by this substrate in CYP3A4 are negligibly small [47], [48].

**Figure 8:**
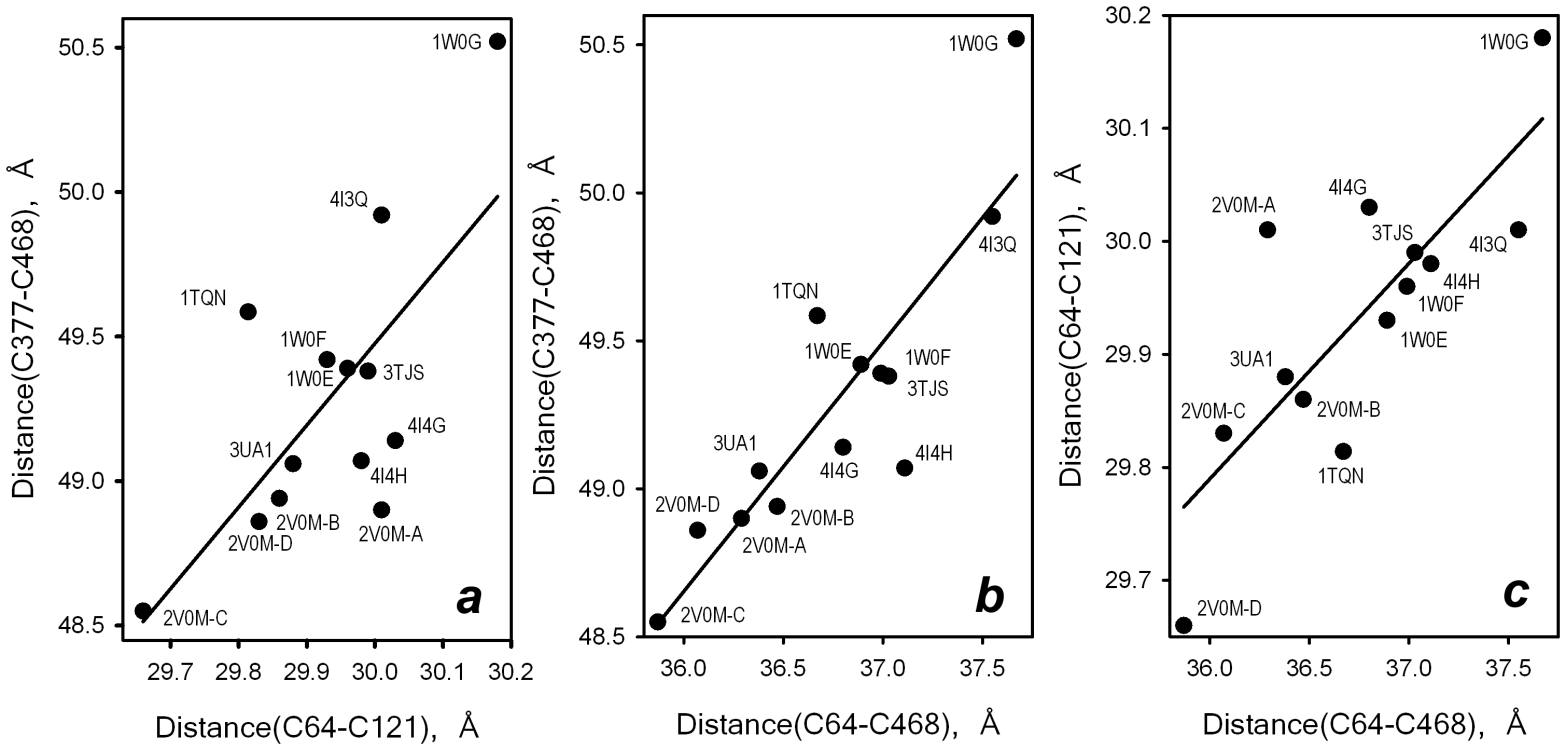
Cross-correlation between inter-residue distances in a set of CYP3A4 X-ray structures. Cross-correlation between the distances between the side chains in Cys_64_/Cys_468_, Cys_377_/Cys_468_ and Cys_64_/Ala_121_ pairs of residues observed in a set of 13 structures of CYP3A4. The distances were calculated between the sulfur atoms of cysteines or beta carbon atom of alanine. The lines represent the results of linear regression of these sets, which are characterized by the square correlation coefficients of 0.50, 0.81 and 0.62 for the plots shown in panels ***a***, ***b*** and ***c*** respectively.

As noted above, the case of bromocriptine and C64/C121-DY/ER represents the only exception from general similarity in the responses of all three pairs of residues observed in our experiments (Fig. 7). This similarity is intriguing, as it may indicate a large-scale conformational transition that involves a concerted movement of the part of the protein that bears the residues Cys_64_ and Cys_377_. Comparative analysis of 17 available sets of molecular coordinates of CYP3A4 (including the coordinates of multiple subunits in asymmetric units of PDB structures 2J0D, 2V0M and 3NXU) suggests that concerted displacement of Cys_64_ and Cys_377_ relative to Ala_121_ and Cys_468_ is commonly observed in CYP3A4. As shown in Fig. 8, the changes in the distances in Cys_64_/Cys_468_, Cys_377_/Cys_468_ and Cys_64_/Ala_121_ pairs reveal a pronounced cross-correlation in all but two known CYP3A4 structures. The two exceptions are the structures of the complexes of CYP3A4 with erythromycin (2J0D) and ritonavir (3NXU), the largest substrates crystallized with the enzyme. Although this correlation indicates that the concerted mobility of Cys_64_ and Cys_377_ relative to Cys_468_ and Ala_121_ may represent a characteristic feature of CYP3A4, the large-scale ligand-induced distance changes observed in our experiments are unprecedented. As seen from Fig. 8, the magnitude of the differences in the inter-residue distances among different X-ray structures (0.5–2 Å) is considerably smaller than the changes observed in our experiments (up to 8.5 Å).

We may infer therefore that the binding of CYP3A4 effectors causes a movement of the beta-domain of CYP3A4 (which includes the sheets β1 and β2 along with the helices A, B, and K′ and the connecting loops [49]), which increases its spatial separation from the remainder of the protein (the alpha-domain). It is notable that most of the known substrate access channels in P450s, (except for the pathways 2c and 2d [40]) may be thought of as located at the interface between the two domains. Concerted movement of the beta domain may be interpreted therefore as a transition between the closed and open conformations of the enzyme. It should be noted, however, that the structural mechanism of this effector-induced opening appears to be different from the transitions inferred from the analysis of known X-ray structures of open and closed conformations of microsomal P450 enzymes. These substrate-induced conformational changes are usually limited to the so-called F-G and B-C regions [2], [4], [40] and considered in terms of adaptation of the size and shape of the substrate binding pocket to the size and shape of the substrate molecule. In contrast, a large-scale concerted movement of the beta-domain of the enzyme evidenced by our results has never been observed. This rearrangement suggests a new type of conformational mobility that is not limited to the above mentioned flexible regions. Most likely this transition would also require concomitant changes in the conformation of the B–C region and the meander loop that connect the beta-domain with the remaining part of the protein (Fig. 1).

A plausible scenario for effector-induced opening of the enzyme may be based on the hypothesis that this transition is caused by the interactions of the effectors with a peripheral binding site located in the proximity of the B/B′, F/F′ loops and the phenylalanine cluster [10], [12], [13]. This site corresponds to a location of the molecule of progesterone detected in the crystallographic dimer of CYP3A4 crystallized in the presence of this steroid (PDB 1W0F [10]). The position of this peripherally-bound progesterone molecule is illustrated in Fig. 1. According to our FRET-based studies of interactions of CYP3A4 with Fluorol 7GA, the binding of this model fluorescent ligand also involves high-affinity interactions at this site [12]. As we discussed elsewhere [13], many structures of CYP3A4 feature a dimer of subunits interacting through their F′ and G′ helices. Similar interactions are also found in the structures of other P450 proteins: 1A2, 21A, 2A6, and 2C8. CYP3A4 and CYP2C8 dimer structures are, however, unique in that the dimer interface forms a cavity that can accommodate ligands. Detailed analysis of crystallographic structures indicates that the high-affinity interactions at this site require oliogomeric protein. This peripheral site appears to represent a ligand-binding cavity formed by the surfaces of two interacting molecules of the enzyme [13]. Importantly, the suggested dimer geometry is compatible with the proposed model of membrane orientation of the enzyme [50], where the hypothetical ligand binding site is located in water-exposed part of the protein in the proximity of the membrane surface.

Important support for the location of the binding site for allosteric ligands at the interface between two subunits was provided by our recent study of activation of membrane-bound CYP3A4 by allosteric ligands, where we demonstrated that such activation of the membrane-bound enzyme by ANF is observed only in enzyme oligomers [13].

Therefore, we hypothesize that the observed effect of ANF, testosterone and cholesterol on CYP3A4 conformation observed in the present study is caused by their binding to the allosteric site at the interface between two molecules of CYP3A4 in oligomers, which are present both in the membrane and in solution. The difference between bromocriptine and 1-PB on the one hand and ANF, testosterone and cholesterol on the other may reflect the differences between the two groups of ligands in their ability to interact with this peripheral site. According to the results of the present study, the interactions at this peripheral site induce a large scale conformational transition that results in more open structure of the effector-bound protein.

## Supporting Information

Figure S1 **Structures of thiol-reactive fluorescent probes used in this study.**
(TIF)

Figure S2 **Structures of CYP3A4 ligands used in this study.**
(TIF)

Figure S3 **Analysis of the content of DYM and ERIA labels in N- and C-terminal fragments of C64/C468-ER/DY digested with BNPS-skatole.** The black and blue lines show the spectra of absorbance of the digested protein before and after passage through a Ni-NTA agarose column, respectively. The spectrum of the protein eluted with 150 mM imidazole is shown in red. The spectra were scaled to correspond to a total concentration of the labels of 1 µM (the amplitudes of the original spectra were in the range of 0.02–0.12 OD units) and smoothed with a moving-frame 5-th order polynomial (frame width of 30 nm). Molar contents of the labels in the samples were determined by approximation with a combination of the prototypical spectra of absorbance of ERIA (λ_max_ = 539 nm) and DYM (λ_max_ = 736 nm). According to this analysis, the treatment of the protein with BNPS-skatole resulted in partial degradation of the DYM chromophore. As a result, the molar ratio ERIA:DYM increased from ∼1 to 5.6. Retention of the C-terminal peptide on the column of Ni-NTA agarose decreased the ERIA:DYM ratio to 1.3. In contrast, the C-terminal peptide eluted with imidazole is characterized by an ERIA:DYM ratio as high as 12.1. According to these results, the distribution of the ERIA label between C-terminal and N-terminal peptides was estimated to be of 2.9:1.
(TIF)

Table S1 **Phosphorescence decay times in labeled CYP3A4 mutants.**
(PDF)

Table S2 **Comparative analysis of steady-state and time-resolved fluorescence data.**
(PDF)
