# Supplementary figures and images for: A Large-Scale Allosteric Transition in Cytochrome P450 3A4 Revealed by Luminescence Resonance Energy Transfer (LRET)
Source: PLoS One. 2013 Dec 23;8(12):e83898. doi: 10.1371/journal.pone.0083898 (PMC3871636; doi:10.1371/journal.pone.0083898)

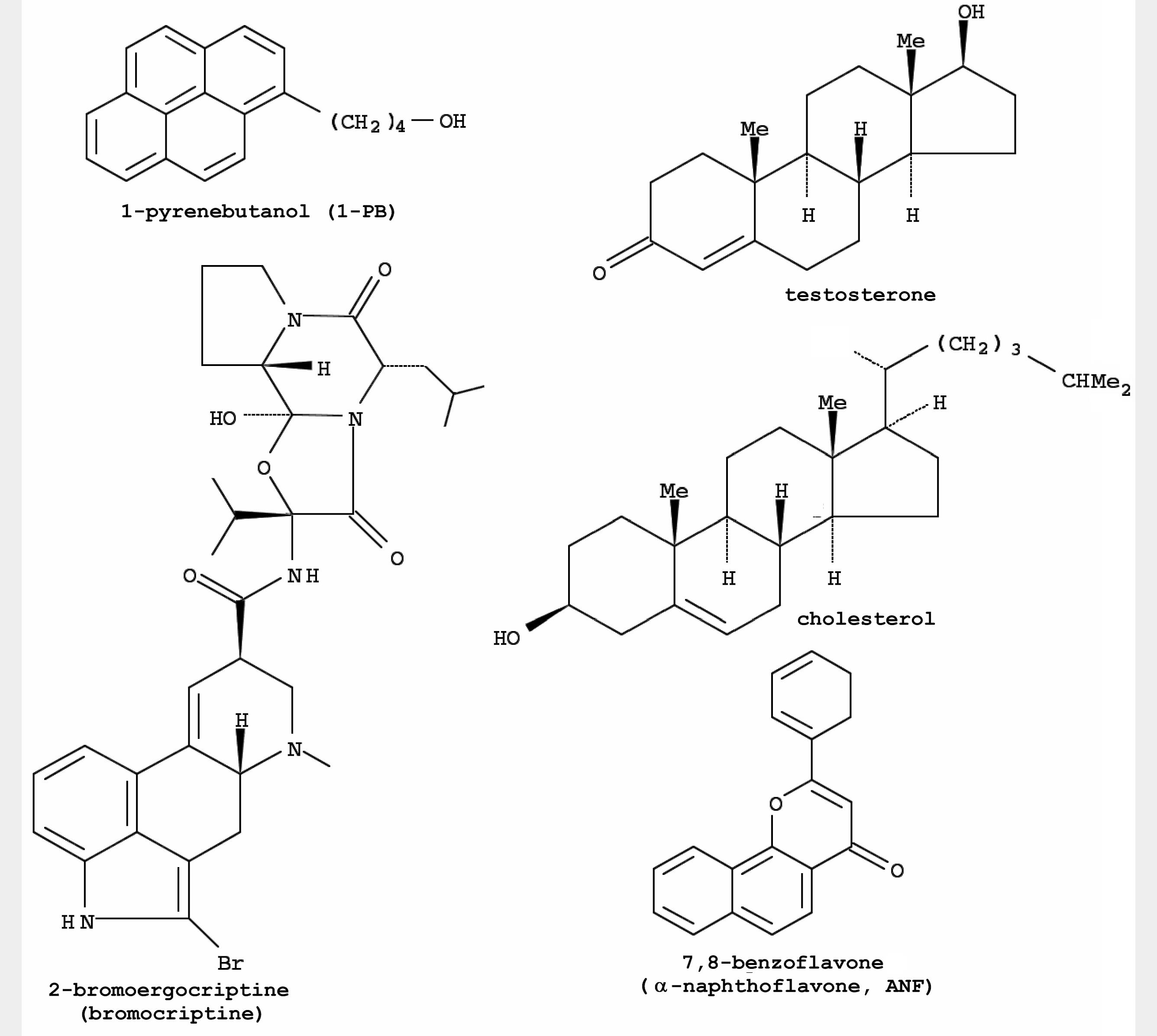

Supplement: Figure S2 — Structures of CYP3A4 ligands used in this study. (TIF) [file pone.0083898.s002.tif]
