# Supplementary material for: A Large-Scale Allosteric Transition in Cytochrome P450 3A4 Revealed by Luminescence Resonance Energy Transfer (LRET)
Source: PLoS One. 2013 Dec 23;8(12):e83898. doi: 10.1371/journal.pone.0083898 (PMC3871636; doi:10.1371/journal.pone.0083898)
Supplement: Table S1 — Phosphorescence decay times in labeled CYP3A4 mutants. (PDF) [file pone.0083898.s004.pdf]

***Table S1*: Phosphorescence decay times in labeled CYP3A4 mutants.**[*]

| Protein | Ligand[a] | $\tau_1$, µs | $\tau_2$, µs | $F_1$, % | $\tau_{av}$, µs |
|---|---|---|---|---|---|
| (C64/C468)-ER/ER | none | 87 ± 7 | 310 ± 22 | 41 ± 8 | 234 ± 4 |
| (C64/C468)-ER/DY | none | 48 ± 6 | 181 ± 7 | 35 ± 4 | 134 ± 3 |
| | 1-PB | 63 ± 2 | 241 ± 5 | 60 ± 8 | 138 ± 4 |
| | ANF | 53 ± 2 | 216 ± 3 | 34 ± 1 | 168 ± 1 |
| | Bromocriptine | 39 ± 12 | 168 ± 2 | 28 ± 5 | 131 ± 5 |
| | Cholesterol | 79 ± 1 | 268 ± 2 | 53 ± 7 | 173 ± 1 |
| | Testosterone | 67 ± 6 | 267 ± 11 | 48 ± 2 | 171 ± 4 |
| (C64/C468)-DY/ER | None | 48 ± 10 | 198 ± 24 | 39 ± 8 | 138 ± 9 |
| | 1-PB | 53 ± 1 | 195 ± 7 | 46 ± 3 | 129 ± 1 |
| | ANF | 54 ± 1 | 231 ± 15 | 45 ± 5 | 154 ± 4 |
| | Bromocriptine | 55 ± 9 | 209 ± 20 | 44 ± 5 | 141 ± 7 |
| | Cholesterol | 59 ± 1 | 221 ± 1 | 43 ± 2 | 152 ± 6 |
| | Testosterone | 69 ± 1 | 247 ± 2 | 39 ± 8 | 176 ± 2 |
| (C377/C468)-ER/ER | None | 69 ± 20 | 272 ± 28 | 44 ± 4 | 182 ± 17 |
| (C377/C468)-ER/DY | None | 25 ± 10 | 129 ± 6 | 20 ± 4 | 108 ± 4 |
| | 1-PB | 45 ± 5 | 149 ± 5 | 30 ± 3 | 118 ± 2 |
| | ANF | 42 ± 10 | 188 ± 18 | 28 ± 5 | 147 ± 8 |
| | Bromocriptine | 25 ± 9 | 129 ± 6 | 20 ± 4 | 108 ± 3 |
| | Cholesterol | 38 ± 1 | 163 ± 8 | 23 ± 5 | 134 ± 3 |
| | Testosterone | 42 ± 9 | 193 ± 5 | 26 ± 7 | 152 ± 12 |
| (C64/C121)-ER/ER | None | 94 ± 10 | 354 ± 19 | 29 ± 4 | 279 ± 8 |
| (C64/C121)-ER/DY | None | 59 ± 7 | 200 ± 15 | 39 ± 5 | 146 ± 5 |
| | 1-PB | 57 ± 1 | 199 ± 12 | 35 ± 1 | 149 ± 9 |
| | ANF | 65 ± 12 | 232 ± 22 | 43 ± 7 | 159 ± 7 |
| | Bromocriptine | 75 ± 13 | 242 ± 15 | 49 ± 5 | 159 ± 2 |
| | Cholesterol | 68 ± 6 | 222 ± 8 | 44 ± 3 | 154 ± 1 |
| | Testosterone | 66 ± 9 | 228 ± 23 | 38 ± 4 | 165 ± 9 |

[*] The values given in the table represent the averages of 3–5 individual measurements, and the ± values show the confidence interval calculated for $p = 0.05$. Experimental curves were approximated with a bi-exponential equation ($\rho^2 > 0.995$).

[a] The concentrations of 1-PB, ANF, BCT, cholesterol, and testosterone used in these experiments were equal to 40 µM, 100 µM, 2.5 µM, 100 µM, and 100 µM, respectively.
