# Supplementary material for: A Large-Scale Allosteric Transition in Cytochrome P450 3A4 Revealed by Luminescence Resonance Energy Transfer (LRET)
Source: PLoS One. 2013 Dec 23;8(12):e83898. doi: 10.1371/journal.pone.0083898 (PMC3871636; doi:10.1371/journal.pone.0083898)
Supplement: Table S2 — Comparative analysis of steady-state and time-resolved fluorescence data. (PDF) [file pone.0083898.s005.pdf]

<u>Table S2</u>. Comparative analysis of steady-state and time-resolved fluorescence data

Protein	Ligand [a]	Steady-state measurements			Lifetime measurements		
		***E*, %**	**R (Å)**	**Δ*R* (Å)**	***E*, %**	**R (Å)**	**Δ*R* (Å)**
C64/C468-ER/DY	none	29 ± 4	39.1 ± 1.5	N/A [b]	43 ± 1	36.3 ± 0.3	N/A
	1-PB	30 ± 1	38.8 ± 0.8	−0.3 ± 0.8	41 ± 1	36.7 ± 0.1	0.4 ± 0.1
	ANF	23 ± 2	41.2 ± 0.9	**2.1 ± 0.9**	28 ± 1	40.4 ± 0.1	**4.1 ± 0.1**
	Bromocriptine	28 ± 5	39.6 ± 1.6	0.5 ± 1.6	44 ± 3	36.0 ± 0.5	-0.3 ± 0.5
	Cholesterol	19 ± 1	42.6 ± 0.7	**3.5 ± 0.7**	26 ± 1	41.1± 0.2	**4.8 ± 0.2**
	Testosterone	20 ± 1	42.2 ± 1.0	**3.1 ± 1.0**	27 ± 2	40.8 ± 0.6	**4.5 ± 0.6**
C64/C468-DY/ER	none	45 ± 2	34.9 ± 0.5	N/A	41 ± 5	36.7 ± 1.0	N/A
	1-PB	41 ± 1	35.8 ± 1.8	0.9 ± 1.8	45 ± 1	35.8 ± 0.1	**-0.9 ± 0.1**
	ANF	29 ± 1	38.8 ± 1	**3.9 ± 1.7**	34 ± 2	38.6 ± 0.5	**1.8 ± 0.5**
	Bromocriptine	43 ± 1	35.1 ± 0	0.2 ± 0.2	40 ± 3	37.1 ± 0.8	0.3 ± 0.8
	Cholesterol	24 ± 1	40.8 ± 2.0	**5.9 ± 2.0**	35 ± 3	38.3 ± 0.7	**1.6 ± 0.7**
	Testosterone	25 ± 1	40.4 ± 1.9	**5.5 ± 1.9**	25 ± 1	41.6 ± 0.3	**4.8 ± 0.3**
C377/C468-ER/DY	none	15 ± 1	44.7 ± 0.6	N/A	41 ± 2	36.8 ± 0.6	N/A
	1-PB	16± 1	44.5 ± 0.7	−0.2 ± 0.7	35 ± 1	38.3 ± 0.3	**1.6 ± 0.3**
	ANF	14 ± 0	45.2 ± 0.1	0.5 ± 0.1	19 ± 4	43.9 ± 2.3	**7.1 ± 2.3**
	Bromocriptine	15 ± 1	44.7 ± 0.1	0.0 ± 0.1	41 ± 2	36.8± 0.4	0.0 ± 0.4
	Cholesterol	14 ± 0	45.2 ± 0.1	0.5 ± 0.1	26 ± 2	41.0 ± 0.6	**4.2 ± 0.6**
	Testosterone	12 ± 2	46.7 ± 0.7	**2.0 ± 0.7**	17 ± 9	45.3 ± 3.0	**8.5 ± 3.0**
C64/C121-ER/DY	none	19 ± 1	42.6 ± 0.3	N/A	48 ± 3	35.1 ± 0.6	N/A
	1-PB	18 ± 1	43.4 ± 0.7	0.8 ± 0.7	46 ± 5	35.4 ± 0.8	0.4 ± 0.8
	ANF	18 ± 1	43.4 ± 0.1	**1.1 ± 0.1**	43 ± 4	36.4 ± 0.5	**1.3 ± 0.5**
	Bromocriptine	17 ± 1	43.6 ± 0.9	1.0 ± 0.9	43 ± 1	36.4 ± 0.1	**1.3 ± 0.1**
	Cholesterol	17 ± 1	43.6 ± 0.5	**1.0 ± 0.5**	45 ± 1	35.8 ± 0.1	**0.8 ± 0.1**
	Testosterone	19 ± 1	43.9 ± 0.5	**1.3 ± 0.5**	40 ± 5	36.9 ± 0.7	**1.9 ± 0.7**

[*] The values given in the table represent the averages of 3–5 individual measurements, and the ± values show the confidence interval calculated for $p = 0.05$. The values of the distance changes were considered significant if their confidence interval falls outside of the −0.5 - +0.5 Å window. These values are emphasized in bold.

[a] The concentrations of 1-PB, ANF, BCT, cholesterol, and testosterone used in these experiments were equal to 40 µM, 100 µM, 2.5 µM, 100 µM, and 100 µM, respectively.

[b] N/A – not applicable
